# Telerehabilitation in Italy During the COVID-19 Lockdown: A Feasibility and Acceptability Study

**DOI:** 10.5195/ijt.2021.6334

**Published:** 2021-06-22

**Authors:** Giada Milani, Giulia Demattè, Matilde Ferioli, Giulia Dallagà, Susanna Lavezzi, Nino Basaglia, Sofia Straudi

**Affiliations:** 1 IIT@UNIFE Center for Translational Neurophysiology, Istituto Italiano DI Tecnologia, Ferrara, Italy; 2 Doctoral Program in Translational Neurosciences and Neurotechnologies, Ferrara University, Ferrara, Italy; 3 Department of Neuroscience and Rehabilitation, Ferrara University Hospital, Ferrara, Italy; 4 School of Physiotherapy, Ferrara University, Ferrara, Italy

**Keywords:** COVID-19, Disability, Physical therapy, Physiotherapy, Technology, Telemedicine, Telerehabilitation

## Abstract

This study examined the feasibility and acceptability of a telerehabilitation program during the COVID-19 pandemic in a sample of adult patients with physical disabilities. Of the twenty-three patients enrolled, 11 agreed to participate in a video-based telerehabilitation program. Barriers and facilitators to the adoption of telerehabilitation were identified and clinical, demographic, and psychological variables were analysed as predictors of success. Age, cognitive reserve, and resilience were significant predictors of satisfaction with telerehabilitation (p<0.05). The telerehabilitation program was perceived as feasible and was well accepted by patients, despite some technology challenges. However, patients who took advantage of telerehabilitation perceived differences in the quality of service and preferred traditional in-person treatment to service delivery via telerehabilitation.

The World Health Organization declared the Severe Acute Respiratory Syndrome - Coronavirus – 2 (COVID-19) outbreak a Public Health Emergency of International Concern on January 30, 2020. The COVID-19 pandemic so overwhelmed Italy's healthcare system that on March 8, 2020, the government implemented containment measures. Italy was the first European Union (EU) country to adopt a precautionary generalized lockdown. This necessitated the adoption of new strategies to continue to provide rehabilitation activities.

One of the most worrying aspects of the COVID-19 pandemic was its impact on persons who were frail and vulnerable, as the virus most negatively affected persons with pre-existing chronic illnesses and physical disabilities (Bartolo et al., 2020; Boldrini et al., 2020a; Leocani et al., 2020). While persons with pre-existing conditions suffered psychiatric distress ascribed to the spread of COVID-19 (Wang et al., 2020; Holmes et al., 2020), little change was observed in symptoms of depression, anxiety, and quality of life during COVID-19 lockdown in patients with progressive multiple sclerosis (Chiaravalloti et al., 2020).

Persons with physical disabilities faced several problems during the pandemic. Communications with healthcare professionals and the delivery of a coordinated multidisciplinary rehabilitation service were interrupted or limited. Also, there was pervasive anxiety that rehabilitation treatments would not be available if the pandemic intensified, or new variants of COVID-19 emerged (Boldrini et al., 2020b).

During the COVID-19 emergency, the delivery of many healthcare services was shifted from in-person treatment to telemedicine (Hilty et al., 2013) and telerehabilitation. Telerehabilitation is an off-shoot of telemedicine that leverages telecommunication technologies to deliver rehabilitation services synchronously and/or asynchronously to patients at a distance, thereby minimizing travel time and costs (Brennan et al., 2010). These technologies, including telemonitoring, teleassistance, among others, and its branches, fall within the realm of digital physical therapy practice (Dantas et al., 2020).

The Italian Society of Physical and Rehabilitation Medicine (SIMFER) webinar held on April 3, 2020 focused attention on telemedicine applications for outpatient rehabilitation (Negrini et al., 2020). Consensus was reached among the physicians that telemedicine could and must be a common practice, especially for screening, distance support, follow-up, and in emergency situations. The experiences described by these professionals were heterogeneous in terms of the populations, intervention types, technologies used, and the applicability of telemonitoring. The perceived potential benefits included an impact on the reduction of isolation, increased motivation and compliance during sessions, and the reduction of barriers for frail and less independent persons to access consultations and treatments. However, some held onto the opinion that telemonitoring will never replace the in-person encounter between the patient and the professional who cares and provides a biopsychosocial holistic, helpful, in-person encounter.

Several barriers (i.e., technology or patient factors) can limit the safe use of telerehabilitation by healthcare institutions. These include data privacy, patient safety, and reimbursement (Negrini et al., 2020; Leochico, 2020). Health care providers’ acceptance of technology is a key element that influences the sustainability and success of telerehabilitation (Brewster et al., 2014; Wade et al., 2014). Other technical barriers that can affect the adoption of telerehabilitation by healthcare institutions include low expertise in using specific hardware and software (Jafni et al., 2019).

Patients also experience technology related barriers to successful participation in telerehabilitation. These may include limited technical resources, absence of devices at home, and slow Internet bandwidth (Negrini et al., 2020; Leochico, 2020). In addition to these technical challenges, other limitations relate to patients’ characteristics and behaviors, given that high levels of physical, emotional, and cognitive efforts are necessary to be engaged in telerehabilitation (Theodoros, 2012). Even though the presence of chronic medical comorbidities and reduced activities of daily living (ADL) were not detrimental to the use of technology in older adults, depressive and anxiety symptoms and other indicators of social integration and social support were significantly associated with Internet use (Choi et al., 2013).

Another possible barrier to the use of telemedicine is patient age (Negrini et al., 2020; Leochico, 2020). Di Giacomo et al. (2013) posited that the ability to use smart technologies may be negatively affected by the impact of aging on cognitive and emotional characteristics. Previous studies described a positive association between computer use and cognitive function in older adults (Czaja et al., 2006; Tun & Lachman, 2010; Fazeli et al., 2013) suggesting that older adults with better cognitive abilities are more likely to use up-to-date technology (Slegers et al, 2012, Wu et al., 2019). Educational level, as part of cognitive reserve, can be another determinant of successful technology use in older adults (Choi et al., 2013) and in patients with Parkinson disease (Dias et al., 2016). Specifically, higher-educated people tended to acquire new knowledge most effectively (Meyer et al., 2015).

The primary aim of this study was to assess the feasibility and acceptability of a telerehabilitation program during the COVID-19 lockdown in a sample of adult patients with physical disabilities. Secondly, given that there is limited information on potential predictors of successful participation in telerehabilitation programs, demographic, clinical, and psychological variables were examined.

## METHODS

This cross-sectional study was performed at the Neuroscience and Rehabilitation Department of Ferrara University Hospital (Italy). The study was approved by the AVEC Ethics Committee. The Strengthening the Reporting of Observational studies in Epidemiology (STROBE) guidelines were followed in this study.

Patients were enrolled if they met the following inclusion criteria: (1) adult female or male; (2) age between 18 and 80 years; (3) presence of physical disabilities; (4) participating in an outpatient rehabilitation program that was interrupted due to COVID-19 government restrictions. We excluded patients with severe cognitive impairment that would have precluded obtaining informed consent and completing questionnaires. Demographic and clinical data were recorded: gender, age, education, nationality, housing situation, presence of a caregiver, employment, diagnosis, number of falls during the lockdown, number of calls to the General Practitioner and other physicians during lockdown, and level of independence in activities of daily living at admission to rehabilitation as measured by the Barthel Index (Bouwstra et al., 2019).

The following questionnaires were administered in-person at readmission to the rehabilitation service to characterize psychological and physical well-being and their association with participation to telerehabilitation:

- Cognitive Reserve Index questionnaire (CRIq) evaluates the cognitive reserve of an individual by means of the compilation of information relating to their entire adult life (Nucci et al., 2012)
- General Self-Efficacy Scale (GSE) evaluates a general sense of perceived self-efficacy with the aim to predict coping with daily frustrations as well as adaptation after experiencing all types of stressful life events (Schwarzer & Jerusalem, 1995)
- Connor and Davidson Resilience Scale (CD-RISC) is a unidimensional self-report scale that measures resilience or how well one is equipped to bounce back after stressful events, tragedy, or trauma (Campbell-Sills & Stein, 2007)
- 12-Item Short Form Survey (SF-12) is a self-reported outcome measure assessing the impact of health on an individual's everyday life (Ware Jr et al., 1996)
- Patient Health Questionnaire (PHQ-9) consists of nine items and assesses different depressive symptoms (Kroenke et al., 2001)
- Beck Anxiety Inventory (BAI) measures the severity of anxiety (Ulusoy et al., 1998)

### TELEREHABILITATION PROGRAM

Beginning on March 16, 2020, each patient was contacted by phone. An offer was made to restart their COVID-19 interrupted rehabilitation program through teleconsultations with a specialized physiotherapist and a medical doctor. The telerehabilitation program aimed to deliver real-time clinical information and physiotherapy services 2–3 times/week according to individual needs during the COVID-19 lockdown; each session lasted approximately an hour. By appointment, an expert physiotherapist contacted the patients by video call at a pre-arranged time to deliver customized exercises. Safety was guaranteed by the physiotherapist's continuous monitoring. The patient could follow verbal prompts and live demonstrations by the physiotherapist, who could correct any mistakes or address any questions and concerns in real-time and share on the screen helpful documents and images. The physiatrist was available in case of specific requests from the client (e.g., pain, drug therapy, etc.). Skype, a free software program owned by Microsoft that allows for free voice and video calls one-to-one or as a group, instant messages, and the sharing files with other people, was employed (Armfield et al., 2015). Skype can be used on a mobile phone, computer, or tablet. The authentication of participants was done at the beginning of every session to ensure the patient's privacy.

### FEASIBILITY AND ACCEPTABILITY ASSESSMENTS

Evaluations of feasibility and acceptability were made at the end of the telerehabilitation program. We defined acceptability as satisfaction with the telerehabilitation program, the perceived strengths and weaknesses of the intervention, and the reasons for non-participation. Feasibility included the rate of participation in the telerehabilitation program, the adherence to the intervention, and familiarity with the technology.

### ACCEPTABILITY

The researchers developed a survey with a list of barriers and facilitators to telerehabilitation to evaluate the acceptability of the telerehabilitation intervention. Participants rated items on a 5-point Likert scale, ranging from 1 (strongly agree) to 5 (strongly disagree). To identify possible obstacles to the completion of this intervention, a list of possible reasons was provided to the patients who did not complete the program, to rate.

### FEASIBILITY

Feasibility was assessed as the rate of participation and adherence to the telerehabilitation program as measured by the percentage of sessions lost, withdrawal, and dropout rate. The System Usability Scale (SUS) was administered to test the usability (Bangor et al., 2008). The Technology Familiarity Scale (TFS) was administered to measure everyday technology in older people and people with a disability (Crotty et al., 2014).

### STATISTICAL ANALYSIS

All continuous variables were expressed as mean ± standard deviation. Between-group differences among patients who either received telerehabilitation or did not, were explored with the Wilcoxon rank-sum test. Correlations between clinical, demographic, and psychological variables and participation/non-participation in the telerehabilitation program or familiarity of technology were tested with the Spearman-Rho test. Finally, a logistic regression analysis was performed including those variables that correlated significantly with the participation in the telerehabilitation program (dependent variable); and a linear regression model was used to underline variables that predicted the familiarity to technology (dependent variable). Statistical significance was set at a level of p<0.05. Statistical analysis was performed using STATA 13.1 software.

## RESULTS

### CLINICAL AND DEMOGRAPHIC CHARACTERISTICS OF THE PARTICIPANT SAMPLE

The telerehabilitation program was proposed to 33 patients who were receiving motor rehabilitation that was interrupted by the COVID-19 lockdown: among them 23 patients were enrolled in the study (57.3±13.3 years, 10 males and 13 females), and 10 patients were excluded (8 patients refused to participate, 1 was a child, 1 had severe cognitive impairments). Eleven of the 23 patients (49.09 ±10.7 years; 4 males and 7 females) took part in the telerehabilitation program. The clinical and demographic characteristics of the two groups are summarized in *Table 1*.

**Table 1:** Clinical and Demographic Characteristics of the Participant Sample

	Mean (SD)	*p* value		
	Telerehabilitation program (N=11)	No-Telerehabilitation (N=12)	Total (N=23)	
**Age (years)**	49.09 (10.72)	64.91 (10.94)	57.34 (13.32)	0.004
**Sex (M/F)**	4/7	6/6	10/13	0.510
**Barthel Index at admission**	76 (15.59)	87.27 (15.71)	81.90 (16.31)	0.054
**Living situation (detached house/apartment)**	3/5	3/6	6/11	0.858
**Diagnosis (neurological disability/orthopedic disability)**	4/7	8/4	12/11	0.146
**Time from the damage to the COVID-19 pandemic (< 6 month/> 6 month)**	7/3	5/5	12/8	0.361
**Live alone/Presence of a caregiver**	2/8	3/8	5/16	0.696
**Employment (not work/work)**	4/5	9/2	13/7	0.081
**Cognitive Reserve Index, total**	116.90 (17.84)	100.25 (13.72)	108.21 (17.64)	0.045

*Note.* Data are presented as mean with SD in parentheses unless otherwise indicated.

At the end of the COVID-19 lockdown (late May 2020), a set of outcome measures were collected to characterize the sample. All enrolled patients returned to in-person rehabilitation for conventional therapy. The group that took part in the telerehabilitation program was not significantly different for clinical and demographic characteristics, except for age (p=0.004), CRIq score (p=0.04) and the performance in activities of daily living at admission to outpatient rehabilitation (p=0.05).

The number of falls at home (p=0.36) and the number of calls to the General Practitioner or other specialists (p=0.94) did not differ between the groups; similarly, there was no significant difference in terms of familiarity with technology (p=0.13). Descriptive analyses showed that all patients reported medium-high levels of resilience (28.6±9.4), high levels of generalized self-efficacy (29.9±8.1), medium levels of anxiety (8.3±5.9) and subthreshold depression (5.6±3.7). Physical (11.1±2.3) and mental self-perceived health (18.08±4.1) were similar across all samples. The psychological and physical profiles of the two groups are summarized in Table 2.

**Table 2:** The Psychological and Physical Well-being at Readmission to Rehabilitation

	Mean (SD)	*p* value		
	Telerehabilitation program (N=11)	No-Telerehabilitation (N=12)	Total (N=23)	
**PHQ9**	5.09 (3.26)	6.16 (4.21)	5.65 (3.74)	0.599
**BAI**	7.09 (5.61)	9.5 (6.33)	8.34 (5.98)	0.323
**GSE**	31.81 (5.43)	28.09 (10.17)	29.95 (8.18)	0.448
**CD-RISC10**	31.63 (8.81)	25.91 (9.61)	28.65 (9.48)	0.280
**TFS**	26.54 (4.94)	22.66 (7.77)	24.52 (6.72)	0.138
**SF-12 PCS**	11.45 (2.84)	10.83 (1.89)	11.13 (2.36)	0.489
**SF-12 MCS**	18.18 (4.66)	18 (3.76)	18.08 (4.12)	0.852

*Note.* Data are presented as mean with SD in parentheses unless otherwise indicated. Abbreviations: PHQ9, *Patient Health Questionnaire*; BAI, *Beck Anxiety Inventory*; GSE, *General Self-Efficacy Scale*; CD-RISC 10, *Connor And Davidson Resilience Scale*; TFS, *Technology Familiarity Scale*; SF-12 PCS, *12-Item Short Form Survey Physical Component Summary*; SF-12 MCS, 12*-Item Short Form Survey Mental Component Summary*

### FEASIBILITY AND ACCEPTABILITY

The overall adherence to the program was high; only one participant missed almost 30% of the sessions due to technical issues. No participants dropped out or withdrew during the program.

**Figure 1:**
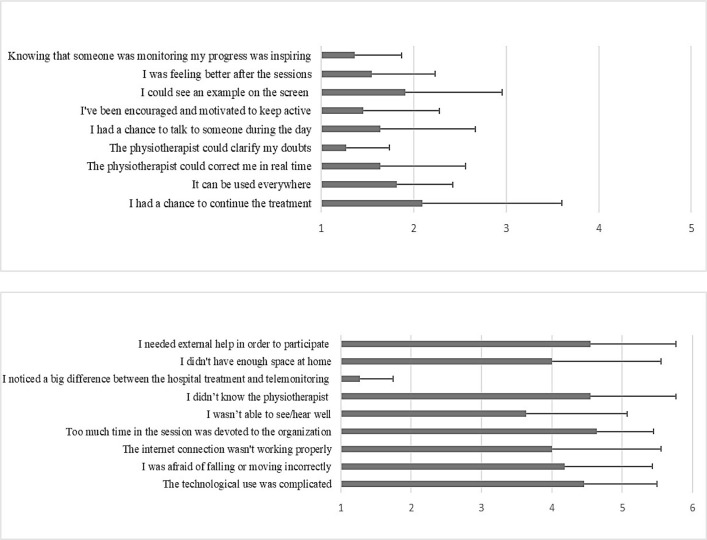
Self-reported Advantages and Disadvantages of Telerehabilitation Program

The group of patients who participated in telerehabilitation reported that the most relevant perceived limitation was the difference between the conventional treatment and telerehabilitation. However, they described the program as beneficial and satisfactory because the physiotherapist could address their questions. They also found it useful knowing that a clinician had monitored their progress; they were therefore encouraged and motivated to stay active. See *Figure 1*.

The patients who took part in the telerehabilitation program reported a good perception of usability as assessed by the SUS (78.6±14.1). The patients who did not take part in telerehabilitation reported their reasons for refusing the treatment were: their perception that the treatment was not useful (n=4); the lack of a suitable Internet connection (n=4); and their inability to use a computer or any other device with an Internet connection (n=4).

### PREDICTORS OF PARTICIPATION IN TELEREHABILITATION AND FAMILIARITY OF TECHNOLOGY

The telerehabilitation program was positively associated with CRIq (rho=0.4427, p=.0506) and negatively associated with age (rho = –0.6248, p = .003) and BI score (rho = –0.5658, p = .009). In the adjusted multiple logistic regression model, age (p=.017) and CRIq score (p=.043) remained a significant predictor for taking part in the telerehabilitation program, contrary to BI (.127). See Table 3. Furthermore, age was negatively correlated with resilience (rho=−0.5722 p=.008).

**Table 3:** Logistic Regression Analysis for Telerehabilitation

Variable	Coefficients		95.0 % Confidence interval		z	Significance
	Beta	Std.error	Lower	Upper		
**Age**	.865	.052	.769	.974	-2.38	.017
**CRIq tot**	1.076	.039	1.002	1.15	2.02	.043
**BI**	.951	.030	.893	1.01	−1.53	.127

*Note.* Abbreviations: CRIq, Cognitive Reserve Index; BI, Barthel Index

**Figure 2:**
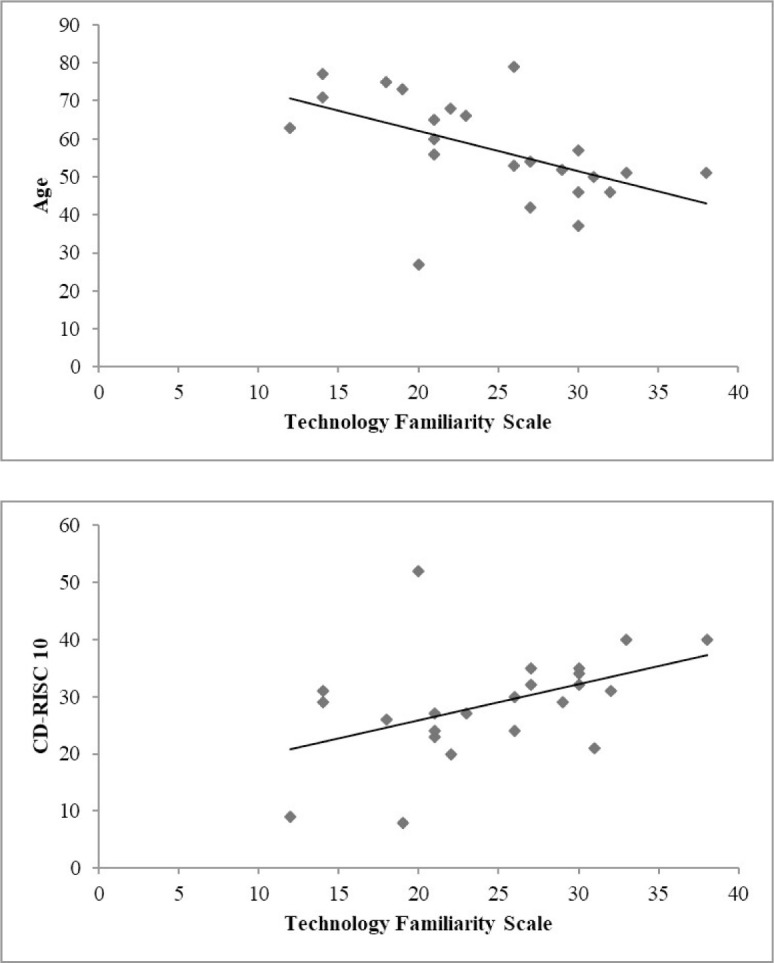
Impact of Age and Resilience on Technology Familiarity

TFS score was positively associated with CRIq score (rho=0.4590, p=.0418) and negatively associated with age (rho=-0.5604, p=.0102) and the number of calls to the General Practitioner or other specialists (rho=0.4802, p=.0321). TFS score was positively associated with GSE score (rho=0.5032, p=.0237) and CD-RISC score (rho=0.5146, p=.0203). TFS score was positively associated with SF-12 PCS score (rho=0.4403, p=.0520) and negatively associated with BAI score (rho=-0.5275, p=.0168). In the adjusted linear regression model, age (p=.009) and CDRISC score (p=.032) remained a significant predictor to familiarity with technology. See *Figure 2* and *Table 4*.

**Table 4:** Linear Regression Analysis for Familiarity of Technology

Variable	Coefficients		95.0 % Confidence interval		t-value	Significance
	Beta	Std.error	Lower	Upper		
**Age**	−.268	.093	−.462	−.074	−2.88	.009
**CD-RISC**	.318	.138	.030	.605	2.30	.032
**BAI**	−.452	.224	−.919	.013	−2.02	.057
**CRIq tot**	.133	.077	−.028	.295	1.71	.101
**GSE**	.291	.171	−.067	.649	1.69	.106

*Note*. Abbreviations: CD-RISC 10, Connor and Davidson Resilience Scale; BAI, Beck Anxiety Inventory; CRIq, Cognitive Reserve Index; GSE, General Self-Efficacy Scale.

## DISCUSSION

During a pandemic, specific actions should be taken to ensure that persons with physical disabilities have equal access to information, health care services, and the support they need to stay healthy and safe. Digital practices offer a safe alternative service delivery model to traditional in-person treatment.

However, some researchers questioned whether physiotherapists were ready to introduce digital practice to provide rehabilitation services during the COVID-19 pandemic period (Dantas et al., 2020). Italy, as is the case for many countries worldwide is in need of additional laws and practical guidelines on how to engage in digital practice, so that required competencies and responsibilities are clearly stated.

The aims of our intervention were to ensure the continuity of rehabilitation while respecting social distancing; to deliver individualized programs to prevent secondary complications; and to maintain the greatest degree of patient autonomy (Boldrini et al., 2020b). Specifically, the study evaluated the feasibility and acceptability of a telerehabilitation program for adults with physical disabilities.

Though Skype is one of the most popular software applications to facilitate video communication (Armfield et al., 2012), little is known about its technical adequacy for telemedicine (Mantokoudis et al., 2013; Bruyneel et al., 2013). Many studies have questioned the advisability of employing the non-subscription version of Skype for clinical purposes, due to its unknown capacity to protect privacy and security. Concerns about the security and privacy of Skype are likely to be a significant barrier to its further uptake in some jurisdictions (Armfield et al., 2012). In our study, sufficient risk-reducing measures were implemented, and no violations were reported regarding privacy, confidentiality, or security.

The patients who participated in telerehabilitation reported a favorable perception of the program's usability. After receiving training, they did not find the technology to be complex and they became comfortable with its use. They also suggested that future users would also quickly adapt to the technology.

Despite these positive reports, the patients who took advantage of telerehabilitation preferred the traditional in-person treatment. They perceived a difference between the in-person hospital and remote treatments in terms of quality of service. Similarly, Cranen et al. (2011) reported that patients with chronic pain were concerned with the lack of therapist in-person contact on their ability to successfully participate in an exercise program, while appreciating the flexibility that telerehabilitation could provide.

There was good adherence to the telerehabilitation program in the current study. All participants completed the intervention and only one patient failed to completely participate due to technical problems related to the Internet connection. Patients described the program as beneficial for the following reasons: the physiotherapist could address patient concerns; knowing that someone monitored the improvement of patients was inspiring; and patients were encouraged and motivated to stay active. Satisfaction with the telerehabilitation was reported as high in two prior studies that compared the satisfaction between telerehabilitation and in-person therapy in stroke patients (Cramer et al., 2019; Piron et al., 2008). Given the overall high levels of satisfaction and the few technical barriers encountered, we can conclude that the telerehabilitation intervention had high acceptability.

Patients who opted not to take part in telerehabilitation reported the most relevant reasons for their treatment refusal were the perception that the treatment was not useful; the lack of a suitable Internet connection; and their inability to use a computer or other devices.

Hamilton et al. (2018) suggested that providers need to systematically support telerehabilitation technology use in their patients. This includes providing positive initial experiences; verbal support during instruction and feedback about performance; support to handle the cognitive demands of using technology; educational support to assist with learning; and motivational and emotional support.

In the current study, familiarity with technology was related to age and resilience; participation with telerehabilitation was associated with younger ages and higher cognitive reserves. Older participants reported lower levels of familiarity with technology than younger people. A possible explanation for this finding is that younger people have had more experience using technology than older people (Bandura et al., 1999). Several large-scale studies found that older adults do not use certain technologies to the extent that younger adults do (Adler, 2006; Madden & Fox, 2006) and suggested that older adults are unable, unwilling, or afraid to use technology.

Familiarity with technology was related to resilience, defined as the ability to readily adapt or recover following change or adversity (Wagnild & Collins, 2009). A person with high level of resilience is able to adapt to new life experiences, restore their balance when faced with difficult situations, and avoid the potentially detrimental consequences of stress. During the pandemic, patients with physical disabilities were forced to newly negotiate their relationships with healthcare providers of telerehabilitation and adapt to previously unfamiliar technologies.

Czaja et al. (2006) found two main personal barriers to technology adoption in the elderly: low self-efficacy and high anxiety regarding computer use. Enhanced training and education may therefore be necessary to inform older users about the benefits of technology use (Czaja et al., 2006). Moreover, prior studies demonstrated a relationship between self-efficacy and technology use and identified issues affecting technology acceptance in older persons and persons with disabilities (Melenhorst et al., 2006; Ziefle & Wilkowska, 2010).

The current findings suggested that both age and cognitive reserve, defined as the set of learning and cognitively stimulating activities acquired by the individual during his life, were uniquely predictive of participation in the telerehabilitation program. Cognitive reserve was evaluated with the CRIq which takes into consideration education, work, and recreational experiences. CRIq is useful to quantify social, cultural, cognitive, and human capital (Nucci et al., 2012). A positive correlation was found between agreement to engage with telerehabilitation and higher cognitive reserve.

Both anxiety and depression had been proposed by Choi et al. (2013) as potential factors influencing Internet use in older adults. The current study, however, did not find a clear relationship between psychological status, physical well-being, and the adoption of the telerehabilitation technology, even though all patients reported medium levels of anxiety and subthreshold depression. Participants in our sample presented with disabilities that were associated with higher levels of anxiety and depression in a prior study (Brenes et al., 2008). Such symptoms can contribute to psychological and physical dysfunction beyond the effects of disability itself with detrimental effects on quality of life (Mitchell et al., 2006).

## LIMITATIONS

This study had several imitations. First, the small sample size limited the significance of the results. However, our sample was unique in that it included a cohort for which outpatient rehabilitation was interrupted during the COVID-19 lockdown. Second, to guarantee the continuity of care during the pandemic, the telerehabilitation program was rapidly implemented with variable training, but without a specific training protocol. Third, the research findings were limited by the cross-sectional design that did not allow for the detection of pre-post variations of selected outcomes. Fourth, information regarding the use of technology was self-reported and digital proficiency was not directly assessed. Lastly, cognitive impairment was an exclusion criteria in this study, limiting the generalizability of the results to adults with cognitive disabilities.

## CONCLUSION

During the COVID-19 lockdown that ceased in-person rehabilitation services, a telerehabilitation program was an adoptable solution, albeit with some associated barriers. While there was good adherence to the telerehabilitation program, and overall positive perceptions of its acceptability and effectiveness, patients preferred “in-person” services.

Several barriers were highlighted. This study showed that younger age and good cognitive reserve can be considered favorable personal characteristics for participation in a telerehabilitation program, whereas resilience and age were more generally associated with familiarity with the technology.
